# Peri-Implant Distal Radius Fracture: Proposal of a New Classification

**DOI:** 10.3390/jcm11092628

**Published:** 2022-05-07

**Authors:** Leonardo Stramazzo, Giuseppe Rovere, Alessio Cioffi, Giulio Edoardo Vigni, Nicolò Galvano, Antonio D’Arienzo, Giulia Letizia Mauro, Lawrence Camarda, Michele D’Arienzo

**Affiliations:** 1 Department of Orthopaedic Surgery (DICHIRONS), University of Palermo, 90133 Palermo, Italy; stramazzoleonardo@gmail.com (L.S.); ale.cioffi90@gmail.com (A.C.); giulio.vigni@gmail.com (G.E.V.); nicologalvano@libero.it (N.G.); michele.darienzo@unipa.it (M.D.); 2 Department of Orthopaedics and Traumatology, Fondazione Policlinico Universitario A. Gemelli IRCCS, Università Cattolica del Sacro Cuore, 00168 Rome, Italy; rovere292@hotmail.com; 3 Department of Orthopaedic Surgery, University of Pisa, 56126 Pisa, Italy; antu84@gmail.com; 4 Department of Physical Medicine and Rehabilitation, University of Palermo, 90133 Palermo, Italy; giulia.letiziamauro@unipa.it

**Keywords:** wrist fracture, plate breakage, plate bending, peri-implant fracture

## Abstract

A peri-implant fracture near the volar plate of the distal radius represents a rarity and can be associated with a mechanical failure of the devices. A literature review was conducted including all fractures that occurred around a volar wrist plate, which could be associated with an ulna fracture. All articles published until December 2021 were considered according to the guidelines presented in the PRISMA Statement. The search was conducted with the PubMed electronic database, Cochrane Database of Systematic Reviews, Medline, Embase, and Google Scholar. Only nine cases of these fractures were reported in the literature. The causes could be due to delayed union/non-union of the old fracture after low energy traumas, high energy trauma in patients with poor bone quality, or hardware mechanical failure. Furthermore, the literature review of peri-implant radius fracture shows different level of radius fracture and types of implant failure. In accordance with these different cases, a new classification of peri-implant fracture of the distal radius is proposed.

## 1. Introduction

Distal radius fractures (DRF) are frequent fractures in the adult population and represent one third of all fractures in the elderly, with an incidence of 190/100,000 per year [1,2]. The surgical management of DRF has undergone extensive changes over the last four decades, from casting to K-wire fixation followed by locked plate fixation. Volar locking plates are being increasingly used for the stabilization of distal radius fractures [3]. Complication rates after volar locking plate fixation of DRF range from 3 to 36% and are widely reported in the literature: sensibility change, tendon irritation or rupture, hardware malfunction, infection, complex regional pain syndrome, and arthritis [4,5]. Mechanical failure of the volar locking plate device is considered to be a rare complication, with failure being defined as plate breakage/bending, screw breakage/loosening, or collapse of articular fragments resulting in intra-articular screw extrusion. Non-prosthetic peri-implant fracture (NPPIF) as a distinct clinical entity is very rare, and only a few articles are reported in the literature [6,7,8,9,10,11,12,13,14]. With the term NPPIF, we referred to an acute bone fracture during a trauma that occurs around implants [15], and it did not include failures of primary fracture fixation such as an implant breakage due to non-union.

In this article, we review all cases of peri-implant radius fracture reported in the literature, and a new classification is proposed according to the different levels of the fracture and the type of plate failure.

## 2. Material and Methods

A review of the literature was performed to investigate all cases of peri-implant radius fracture according to the guidelines presented in the PRISMA Statement (Preferred Reporting Items for Systematic Reviews and Meta-Analyses) [16]. All cases included were of peri-implant radius fractures that occurred around a previous fixation of a wrist fracture with a volar plate in patients over 18 years of age. Cases with an ulna fracture associated with the radius fracture were also considered, including only the detailed cases described regarding the fracture and its treatment. The search was conducted with the PubMed electronic database, Cochrane Database of Systematic Reviews, Medline, Embase, and Google Scholar. The search was conducted including all studies published until December 2021. The following MeSH entries were used for research articles: peri-implant wrist fracture, breakage plate, bending plate, fracture plate wrist, radius hardware failure, radius refracture. All journals were included, and all relevant studies were considered for this study. No filters were applied to the search strategies, and only papers published in English were considered for inclusion. Three reviewers (L.S., A.C., and L.C.) independently conducted the research. Papers were initially identified based on the title and abstract. Investigators separately reviewed the abstract of each publication and then performed an accurate reading of all extended papers to minimize bias. The researchers (L.S., A.C.) checked all the references from the identified articles in order to not miss any relevant study.

## 3. Results

**Figure 1:**
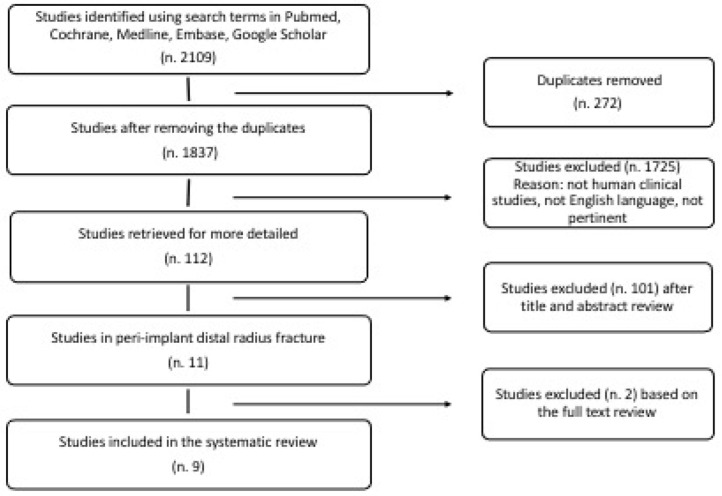
Flow diagram that describes the number of studies identified, included, and excluded as well as the reasons for exclusion.

Nine manuscripts fulfilled the inclusion criteria and were included in the review [6,7,8,9,10,11,12,13,14] (Table 1 and Figure 1). The articles included in this review were all case report studies (nine patients). In four patients, hardware failure occurred after small efforts or low energy traumas [6,7,8,12]; in four cases, the new fracture occurred a few months after the fixation and along the old line of fracture [6,7,8,14]. Loss of reduction with implant failure was presented in two cases without trauma or particular efforts [13,14]. In one case, the fixation failure occurred after seven days [13]. The causes of these fractures were due to delayed union/non-union (promoted by patient’s comorbidity or by smoking habit) or to implant design failure (high rigidity of the hardware, number and direction of proximal and distal locking screws). In four patients, the fractures occurred years later from the primary surgery when the old fracture was already healed [9,10,11,12]. In this case, fractures occurred following a high energy trauma (three patients) and in a patient affected by osteoporosis and poor bone quality.

Regardless of the type of trauma, the condition of the plate or site of the fracture can vary. In fact, in three cases, the plate was bent, and in all these cases the fracture occurred under the plate. One patient was treated with close reduction and plate alignment [8], while two other patients were treated with plate removal and a new plate placement [9,10]. One of these cases was associated with a compound ulna fracture that did not require surgical fixation [10]. In four cases, the new fracture occurred along the old line of fracture with the loosening of the previous reduction [6,7,13,14]. In all these cases, the plate was broken and different treatments were reported. In three cases, the broken plate was removed and a new reduction with a new plate was performed [6,13,14]. In the other case, a closed reduction and splint immobilization was performed due to comorbidities of the patient [7]. In the last two cases reported in the literature, the plate was whole and the fracture occurred proximally to the plate and was associated with an ulna fracture; in both cases, the authors opted for a substitution of the old implant with a longer volar plate and synthesis of the ulna fracture with a plate [11,12].

## 4. Discussion

Peri-implant distal radius fractures are rare, but their number will go up due to the increased use of volar plates for wrist fractures fixation [17]. The causes of these different fractures include patient factors (comorbidity such as osteoporosis, smoking), biological factors (complex fractures), and mechanical factors (no bone graft, unfilled screw holes, and insufficient immobilization). In a recent systematic review of 52 articles, Yamamoto et al. analyzed the hardware removal and complication rate of using a volar locking plate for a distal radius fracture; they did not specifically talk of peri-implant fractures or damaged plates, but they generally reported a hardware problem in 14% of cases and refracture in 1% [18]. A recent study on early postoperative complications that occurred in 594 patients with a distal radius fracture treated with a volar locking plate and a minimum 1-month evaluation reported only two cases of peri-plate fracture. They occurred proximally to the plate, and any cases of plate breakage or bending were reported [19].

Based on the type of fracture, it will be necessary to investigate the precise position of the fracture in relation to the plate and its condition. Further, is mandatory to evaluate the location of the previous wrist fracture in order to plan the surgical treatment. In 2017, Chan and coll. proposed a classification of non-prosthetic peri-implant fractures (NPPIF), which considers the type of implant (nail or plate), the type of fracture (close or far to the implant) in any part of the body, and the healing of the old fracture. However, the authors did not consider the failure of the old plate such as breakage or mobilization [15].

**Figure 2:**
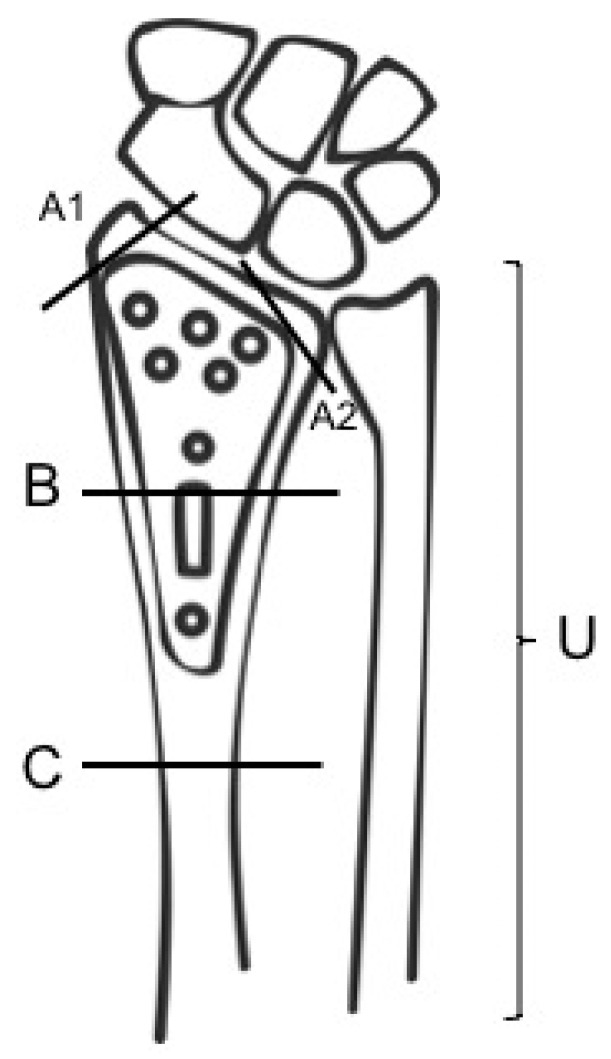
Michele D’Arienzo Classification of perisynthesic fractures of the distal radius with different levels.

According to the literature review, a classification for the peri-implant wrist fractures was proposed by the senior author (Michele D’Arienzo), and it can be comparable to Duncan’s classification for periprosthetic hip fractures [20,21,22]. This new classification, which we prefer to define “perisynthesic” by analogy with periprosthetic, contemplates three types: A, B, and C, based on the location of the fracture and the type of plate failure (bending or breakage) (Figure 2). We call A1 fractures those of radial styloid and A2 those of the medial part of distal epiphysis of the radius. The type B fractures are radius ones that occur under the plate, and we distinguish them as type B1 if there is a bending of the plate and B2 if the plate is broken. In type C, radius fracture occurs proximally to the plate and we call them C1 or C2, depending on whether they occur within 3.5 cm from the plate or beyond 3.5 cm. If there is an association with a fracture of the ulna, we associate the letter U with these abbreviations (e.g., A1U or B2U).

According to this new classification, we propose the following therapeutic algorithm: for A1 and A2 fractures, we suggest non-invasive treatment in the case of compound fractures or surgical treatment in relation to the type of displacement (K-wires, screws). For type B1 and B2 fractures, it is necessary to replace the old plate with a new implant, though in the literature it is also reported that two cases were successfully treated with closed reduction and immobilization [7,8]. In the case of C1 fractures, it is necessary to replace the old plate with a longer plate, while in C2 the fracture is located at a distance such from the old implant that there is enough space to insert a new plate proximally. These must eventually be associated with a reduction and synthesis of the ulna according to level and eventual displacement of the fracture.

## 5. Conclusions

A peri-implant fracture near the volar plate of the distal radius represents a very rare injury, but, considering the growing use of plates, its frequency will probably increase. A trauma of high energy associated with poor bone quality (osteoporosis) can determine a re-fracture around the plate, even if the previous implant was stable, as a low energy trauma in delayed union/non-union fracture can do it too. In addition, the new trauma can cause the bending or the breakage of the plate. In the literature, there is not an exhaustive classification for these types of lesions, and our classification describes a specific point of perisynthesic fracture and the treatment algorithm.

## Figures and Tables

**Table 1:** Literature review of perisynthesic fractures of the distal radius with their main features.

Authors Year of Publication	Years Old	Sex	Time from Primary Implant	Type of Trauma	Site of the Fracture	Plate Condition	Ulnar Fracture	Neuro-Vascular Compromise	Treatment
De Baere et al., 2007	58	F	3.5 months	Effort	Loss of previous reduction	Broken	No	No	Substitution of old implant with new plate
Yukata et al., 2009	82	F	3 months	Effort	Loss of previous reduction	Broken	No	No	Splint
Imade et al., 2009	56	M	7 days	Unknown	Loss of previous reduction	Broken	No	No	Substitution of old implant with new plate
Geurts et al., 2012	78	F	6 months	Accidental fall	Under the plate	Bent	No	Yes	Close reduction and alignment of the plate
Khan et al., 2012	30	M	2 months	Unknown	Loss of previous reduction	Broken	No	No	Substitution of old implant with new plate
Lucke-Wold et al., 2016	73	F	3 years	Trafficaccident	Under the plate	Bent	Yes	No	Substitution of old implant with new plate
Kanji et al., 2017	50	M	11 years	Trafficaccident	Under the plate	Bent	No	Yes	Substitution of old implant with new plate
Barrera-Ochoa et al., 2017	34	M	9 years	Traffic accident	Proximally to the plate	Whole	Yes	No	Substitution of old implant with a longer volar plate and a plate for the ulna
Stramazzo et al., 2020	61	F	4 years	Accidental fall	Proximally to the plate	Whole	Yes	No	Substitution of old implant with a longer volar plate and a plate for the ulna

## Data Availability

Not applicable.
